# Novel affibody molecules targeting the AXL extracellular structural domain for molecular imaging and targeted therapy of gastric cancer

**DOI:** 10.1007/s10120-024-01568-5

**Published:** 2024-12-07

**Authors:** HuiHui Zhang, Maolin Zheng, YiQi Cai, Saidu Kamara, Jun Chen, Shanli Zhu, Lifang Zhang

**Affiliations:** 1 Institute of Molecular Virology and Immunology, Department of Microbiology and Immunology, School of Basic Medical Sciences, Wenzhou Medical University, Wenzhou, 325035 Zhejiang People’s Republic of China; 2 Department of Gastrointestinal Surgery, The First Affiliated Hospital of Wenzhou Medical University, Wenzhou Medical University, Wenzhou, 325035 Zhejiang People’s Republic of China

**Keywords:** Affibody molecules, Gastric cancer, Axl receptor tyrosine kinase, Molecular imaging, Targeted therapy

## Abstract

Gastric cancer (GC) has a poor prognosis and high mortality because it is often diagnosed at an advanced stage. Targeted therapeutics are considered an important class for advanced GC treatment. However, the fewer effective therapeutic targets and the poor coverage of the GC population limit the use of GC targeted therapies. Recent research suggests that the AXL receptor tyrosine kinase (AXL) plays an vital role in the survival and proliferation of GC cells, and blocking AXL pathway may be an effective strategy for targeted therapies. On the other hand, the affibody molecule, with its small size and faster penetration of tissue, has great potential in tumor imaging and targeted therapy. In this study, we report the novel AXL-binding affibody molecules (Z_AXL_:239) screened by a phage-displayed peptide library. The Z_AXL_:239 could specifically bind and interact with AXL proteins in vitro and in vivo, as demonstrated by surface plasmon resonance, co-immunoprecipitation, immuno-fluorescence co-localization, and near infrared fluorescent imaging. In addition, Z_AXL_:239 affibody molecules could significantly inhibit the proliferative activity and induce apoptosis of AXL-positive GC cells by decreasing the phosphorylation levels of the PI3K/AKT1 and MEK/ERK pathway, leading to the suppression of the downstream nuclear protein c-myc. Moreover, Z_AXL_:239 was found to have significant anti-tumor effects in AXL-positive GC transplantation tumor nude mouse models. In brief, we provide strong evidence that the novel Z_AXL_:239 affibody molecules have great potential as a potent tumor-specific molecular imaging and targeted therapeutic agents for GC.

**Supplementary Information:**

The online version contains supplementary material available at 10.1007/s10120-024-01568-5.

## Introduction

Gastric cancer (GC) is one of the most common and lethal malignancies worldwide [1], with approximately half of GC patients are already at an advanced stage when they are first diagnosed [2]. Although advanced GC patients treated with comprehensive treatment including surgical resection, chemotherapy, immunotherapy and targeting therapies, patients often have a poor prognosis due to locoregional recurrence or distant metastasis [3]. Targeted therapies are increasingly being used to treat advanced GC and show promise for improving patients outcome. However, fewer effective therapeutic targets and poor population coverage in GC limit the use of targeted therapies [4]. Therefore, there is an urgent need to develop small molecule imaging probes and targeted therapeutics to improve clinical outcome of GC patients.

Currently, targeted therapies based on monoclonal antibodies are considered an important class of targeted therapies treatment to improve survival and reduce toxicity for GC patients. But the progress of targeted therapy in GC lags significantly behind other solid tumors such as lung cancer, breast cancer, and colorectal cancer [5]. Effective targeted therapies for GC are relatively limited and often rapidly become drug resistant. For example, anti-HER-2 therapy is the first-line targeted therapy [6], but less than 20% of patients can benefit from anti-HER-2 antibody treatments [7] and face some problems such as insufficient drug penetration and rapid drug resistance [8]. Thus, relevant deficiencies of target therapy remain to be further addressed.

Affibody molecules, a class of small affibody proteins, were introduced to biotechnology as an alternative to antibodies 30 years ago [9], generated from a combinatorial library of the triple helix Z-domain framework derived from staphylococcal protein A [10, 11]. Using phage display methods, affibody molecules with specific affinity for a target can be eluted from libraries constructed by combinatorial randomisation of amino acid residues in the Z-domain backbone [12]. The affibody molecules, which are only 1/20th the molecular weight of antibodies, enable efficient tumor penetration, resulting in rapid tumor localization, rapid clearance from the bloodstream and other unspecified tissues, and hold great promise in molecular imaging and molecularly targeted therapies for tumours [13]. As of today, there are more than 50 types of affibody molecules in development, such as HER2 [14], epidermal growth factor receptor (EGFR) [15], melanoma antigen A3 (MAGE-A3) [16], and HPV 16/18 E7 [17, 18].

AXL is a member of the TAM family consisting of extracellular, transmembrane, and intracellular domains [19], with growth arrest specific protein 6 (GAS6) acting as a ligand binding to the extracellular structural region of AXL [20]. Upon binding to the Gas6, the AXL receptor undergoes homodimerization or heterodimerization followed by trans autophosphorylation within the structural domain of the intracellular kinase [21], which activates downstream signalling pathways [22–24]. GAS 6/AXL signaling pathway plays an essential role in tumor cell survival, migration, proliferation, epithelial–mesenchymal transition (EMT), inhibition of apoptosis and drug resistance [25, 26], which promising a potential targeted for treatment [27–29].

Recent studies have suggested that AXL is often highly expressed in GC and may plays an vital role in the survival and proliferation [26, 30–33]. In our study, we showed that AXL is upregulated in GC tissue and that its high expression is associated with a poorer prognosis in patients. Then, we screened and characterized potential novel AXL-binding affibody molecules (Z_AXL_:239) that exhibit specific binding interactions for AXL proteins in vitro and in vivo. In addition, we verified Z_AXL_:239 affibody molecules, which significantly inhibited the proliferative activity of AXL-positive GC cells and found significant anti-tumor effects in AXL-positive GC transplantation tumor nude mouse models, which may be through MEK/ERK signaling pathway. In summary, the novel Z_AXL_:239 affibody molecules will hopefully to be molecular imaging and targeted therapeutic agents in clinical to improve the outcome of GC.

## Results

### Upregulation of AXL expression predicts poor prognosis in GC

**Fig. 1:**
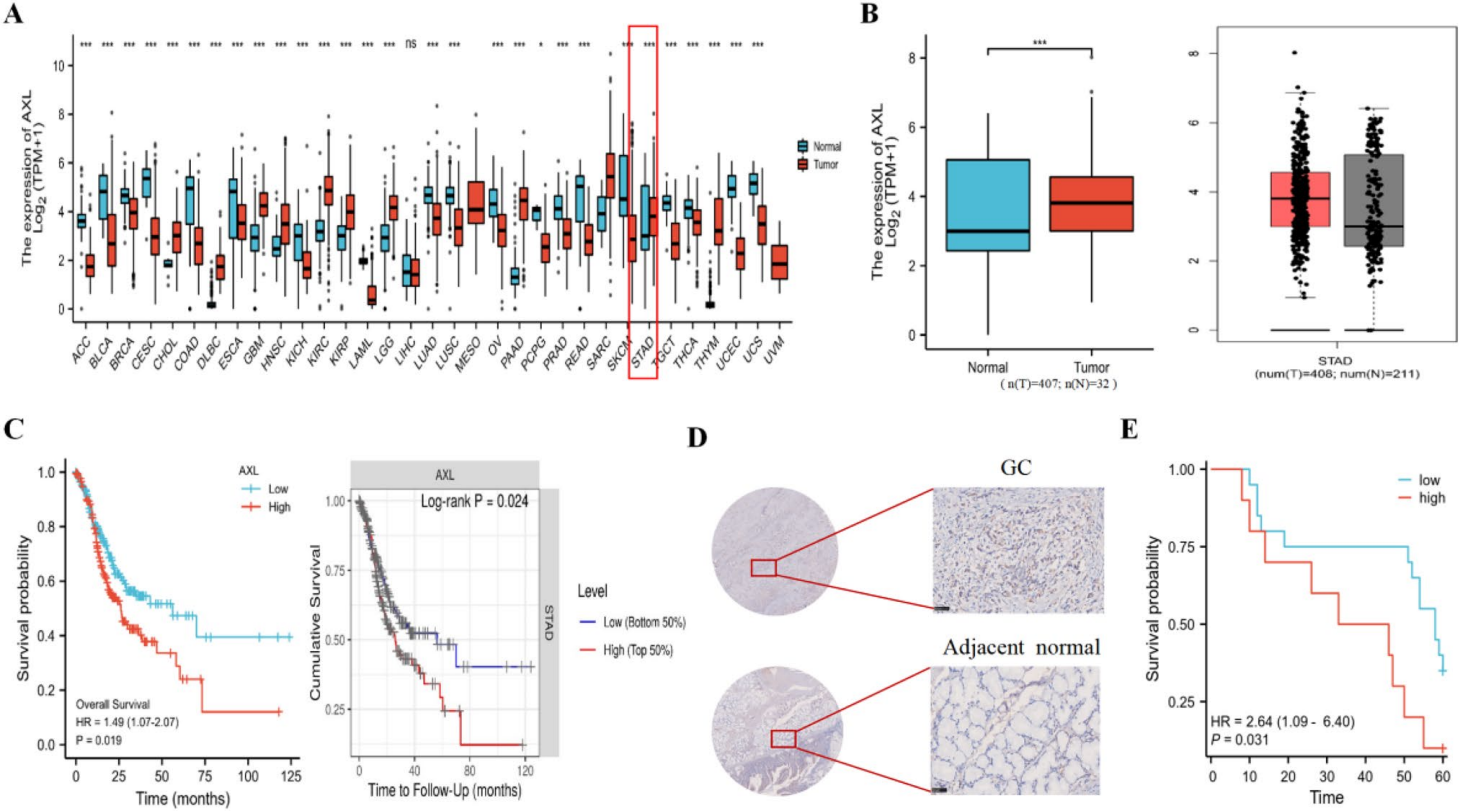
Expression of AXL in gastric cancer. **A** AXL expression in pan-cancer was investigated with the TCGA database. **B** Increased expression of AXL in gastric cancer compared to normal tissues in the GEPIA and TCGA database. **C** Correlation between AXL and prognosis of gastric cancer in TIMER and Kaplan–Meier plotter database. **D** Intensity score (H-score) of AXL expression on gastric cancer and adjacent tissue microarray (*n* = 30). Different tissue regions on the microarray were analyzed using the H-score, which depends on the percentage of positive cells to all cells in the section and the intensity of the staining. **E** Correlation between AXL and prognosis of gastric cancer using tissue microarray (*n* = 30). *p < 0.05; **p < 0.01; ***p < 0.001

We collected and analysed RNA data from normal (*n* = 32) and cancer patients groups (*n* = 375) in the TCGA database to investigate AXL expression in pan-cancer (Fig. 1A,B). Based on the AXL RNA-Seq data in gastric adenocarcinoma tissues, the GC patients were divided into a high expression group (*n* = 188) and a low expression group (*n* = 187) with a cutoff of 4.80 transcripts per million. We found that AXL expression was associated with worse overall survival in GC (Fig. 1C), which showed no association with clinicopathological characteristics (Table S1). We further validated the upregulation expression of AXL between GC tissues over adjacent tissues (Fig. 1D, n = 30), and high expression of AXL group with worse OS by IHC staining analysis (Fig. 1E). Multivariate COX regression analysis suggested that AXL expression, as well as gender and pathological stage, were independent risk factors for GC patients prognosis (Table S2). Our results suggest that AXL expression is a reliable prognostic factor in GC patients.

### Selection and expression of the AXL-binding affibody molecules

**Fig. 2:**
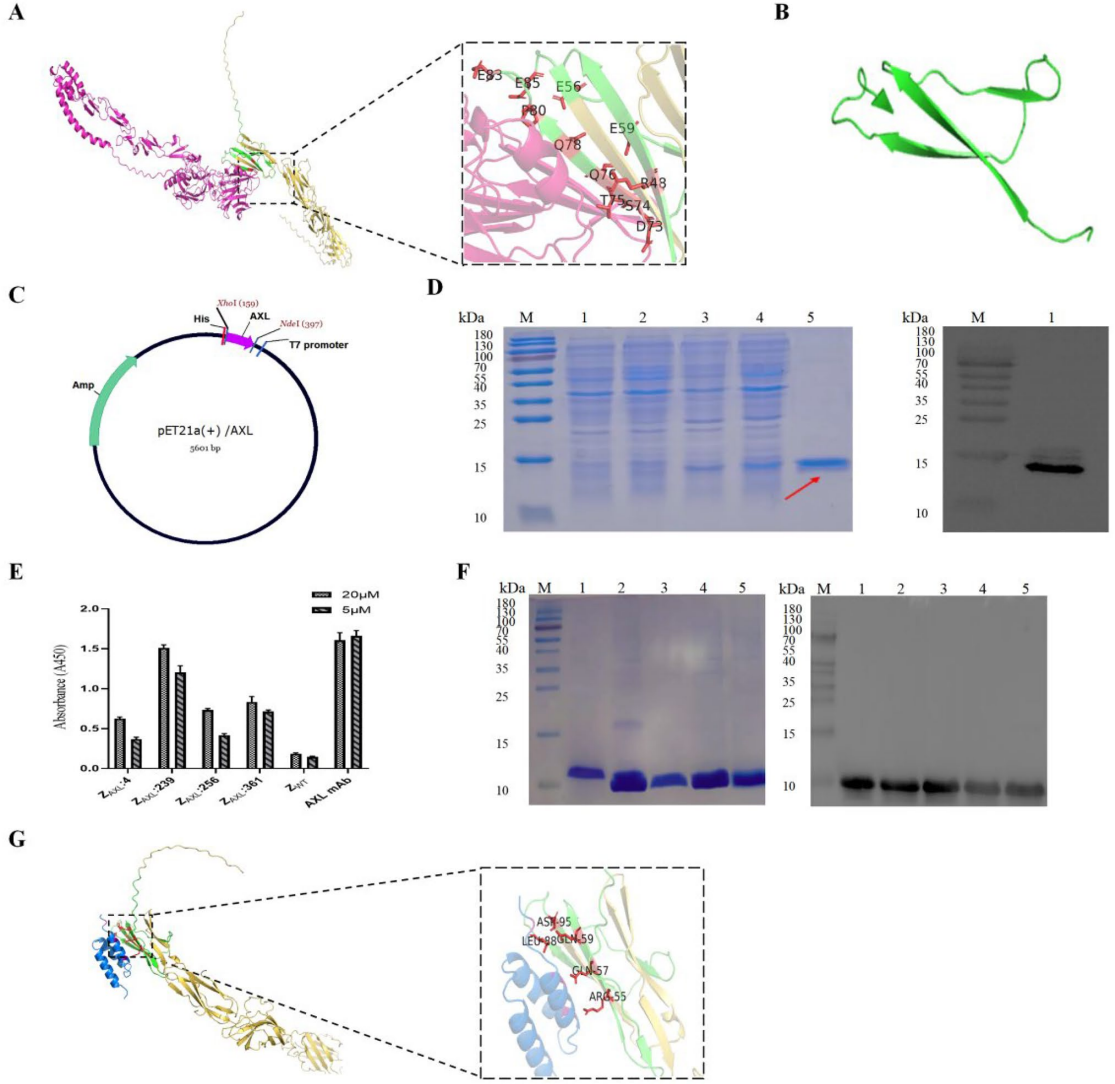
Screening, preparation, purification, and identification of AXL-binding affibody molecules. **A** A model representation of the GAS6/AXL extracellular structural domain 1:1 co-complex is shown, with the GAS6/AXL contact sites labelled. **B** A model representation of amino acids 26–96 of the AXL. **C** Schematic structure of pET21a( +)/ ZAXL affibody recombinant plasmid. **D** Coomassie Brilliant Blue staining SDS-PAGE gel of the recombinant proteins. M, protein ladder; 1, Empty E.coli BL21 (DE3); 2, E.coli BL21 (DE3) transformed with pET21a( +) empty vector; 3, E.coli BL21 (DE3) transformed with pET21a( +)/the recombinant AXL without induction; 4, E.coli BL21(DE3) transformed with pET21a( +)/the recombinant AXL induced by 1 mM IPTG for 6 h; 5, pET21a( +)/The purified the recombinant AXL were analysed by SDS-PAGE, and confirmed by Western blotting with anti-His-tag mouse monoclonal antibody. **E** Screening and selection of four AXL-binding affibody molecules. **F** Coomassie Brilliant Blue staining SDS-PAGE gel of the recombinant proteins. M, protein ladder; 1–5, E. coli BL21(DE3) transformed with pET21a( +)/Z_AXL_:4, pET21a( +)/Z_AXL_:239, pET21a( +)/Z_AXL_:256, pET21a( +)/Z_AXL_:361 and pET21a( +)/Z_WT_ plasmid induced by 1-mM IPTG for 6 h, respectively. The purified ZAXL affibody molecules were analyzed by SDS-PAGE, and confirmed by Western blotting. **G** Molecular docking model of Z_AXL_:239 affibody molecules to AXL

**Fig. 3:**
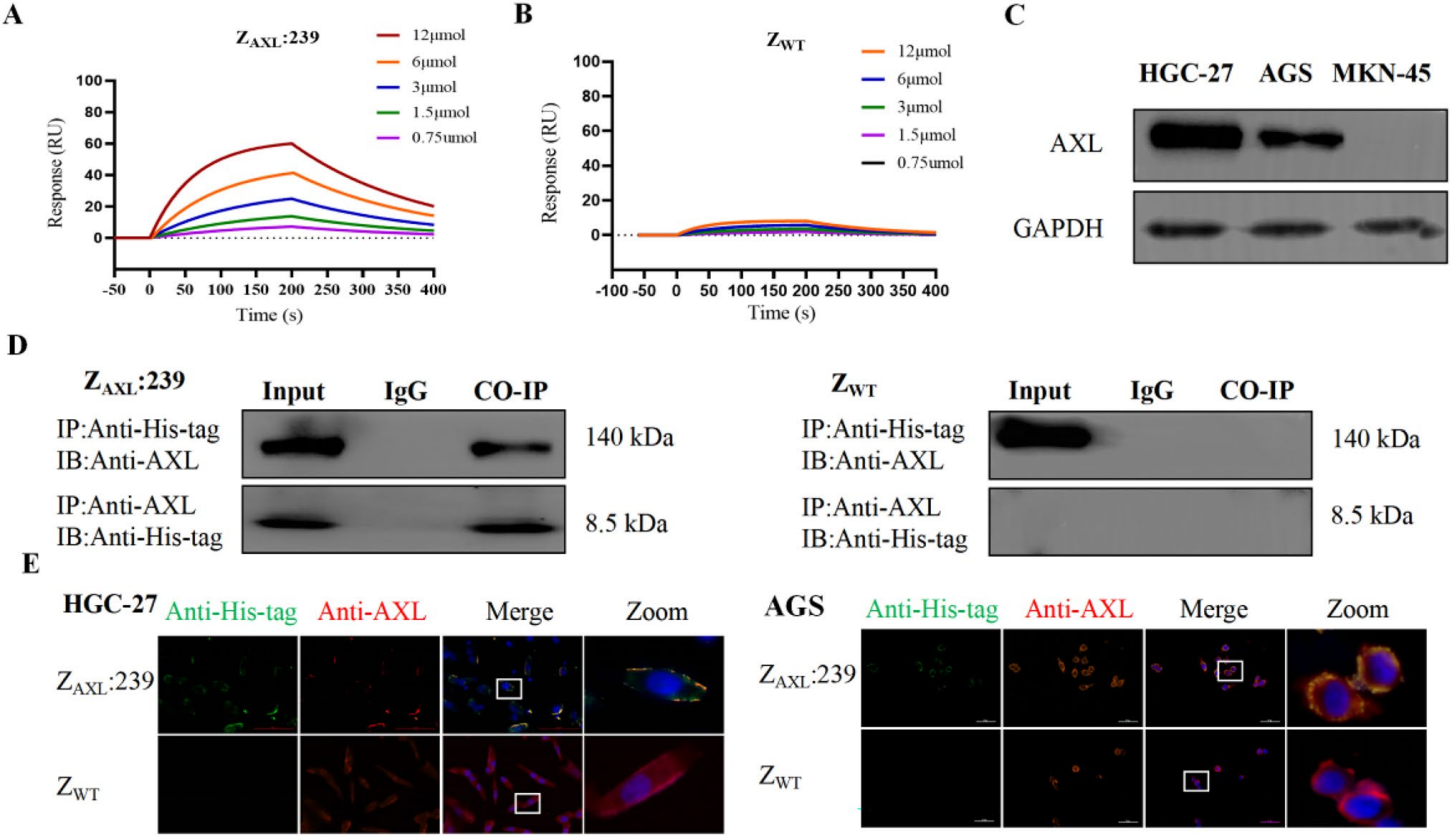
In vitro affinity and binding specificity of Z_AXL_:239 affibody molecules. **A**, **B** Biosensor assays. Representative binding sensorgrams showing the interaction of affibody molecules with immobilized recombinant AXL protein. Binding of 0.75, 1.5, 3, 6, 12 μM Z_AXL_:239 (**A**) and Z_WT_ (**B**) to recombinant AXL protein on the sensor chip was analyzed by SPR-based binding assay. The unselected original affibody scaffold molecule Z_WT_, which has no binding affinity for AXL, was used as a control. **C **The expression of AXL in gastric cancer cell lines was analyzed by Western blotting. **D** Co-immunoprecipitation of Z_AXL_:239 affibody molecules and native AXL protein in HGC-27, AGS and MKN-45 cells. **E** immunofluorescence co-localization of Z_AXL_:239 affibody molecules and native AXL protein in HGC-27, AGS and MKN-45 cells

As shown in Fig. 2A, analysis of the molecular docking model and interactions of GAS6 with the AXL extracellular structural domain suggests that GAS6 binds to amino acids 26–96 of the AXL (Fig. 2B), which was selected as the recombinant target protein for screening (Fig. 2C). Three potential affibody molecules, Z_AXL_:4, Z_AXL_:239 and Z_AXL_:361, showed strong affinity with AXL protein (Fig. 2E). SDS-PAGE electrophoresis results showed that the molecular weight of the AXL-binding affibody was approximately 8.5 kDa, and the purified product had a purity of approximately 90% (Fig. 3F). Detailed protein expression and purification methods are described in later methods. Taking into account affinity and expression efficiency, Z_AXL_:239 was finally selected for subsequent experiments.

The recombinant GAS6 protein was similarly expressed and purified for subsequent experiments. Western blotting results showed that recombinant AXL can specifically interact with the ligand GAS6 (Fig. S2).

### Molecular docking model of Z_AXL_:239 to AXL

As shown in Fig. 2G, the binding site of the AXL extracellular structural domain to the Z_AXL_:239 affibody was simulated and compared with the binding site of GAS6 to the AXL extracellular structural domain using AlphaFold2. Analysis of the molecular docking models indicates that both Z_AXL_:239 and GAS6 bind to amino acids 26–96 of the AXL extracellular structural domain.

### Biosensor-binding analyses of Z_AXL_:239 intertion with AXL

Purified Z_AXL_:239 affibody was validated by SPR to verify real-time biological interactions with the recombinant AXL protein. Different concentrations of affibody proteins were injected onto a chip containing immobilized the recombinant AXL protein on a BIAcore T-100 biosensing device, and the results showed that the resonance response signals were dependent on the concentration of Z_AXL_:239 affibody molecules (Fig. 3A). In contrast, no effective resonance signal could be detected at different concentrations of Z_WT_ affibody molecules (Fig. 3B).

Further kinetic analysis showed that Z_AXL_:239 had a dissociation equilibrium constant (KD) of 3.19E-06 mol/L, significantly lower than that of Z_WT_, which was 7.96E-02 mol/L (Table 1). The above results indicate that the Z_AXL_:239 binds to the recombinant AXL protein with high affinity.

**Table 1:** Kinetic data analysis of surface biosensors

	Ka (1/Ms)	Kd (1/s)	KD (M)
Z_AXL_:239	3.92E + 4	1.259E-1	3.196E-6
Z_WT_	3.504E-2	2.795E-3	7.96E-2

*Ka* Association rate constant, *Kd* Dissociation rate constant, *KD* Dissociation equilibrium constant

### High binding specificity interacted of Z_AXL_:239 with native AXL

HGC-27 and AGS were considered to be AXL-positive cell lines, whereas MKN-45 was considered to be AXL-negative cell (Fig. 3C). Co-immunoprecipitation experiments confirmed that endogenously expressed AXL proteins directly and specifically interacted with the Z_AXL_:239 affibody (Fig. 3D).

Moreover, we examined the binding of Z_AXL_:239 molecules to native AXL proteins in the cell membrane by immunofluorescence co-localization assay. In HGC-27 and AGS cells, incubation of the cells with Z_AXL_:239 showed that resulted in the simultaneous appearance of red (labeled anti-AXL antibody) and green (labeled anti-His-tag antibody) fluorescence signals, which co-localized at the same position of the cell membrane. Meanwhile, there was no visible FITC staining on the membranes of AXL-overexpressing cells incubated with Z_WT_ molecules and AXL-negative cells (Fig. 3E).

### Tumor-targeting ability of Z_AXL_:239 affibody in mice model

Z_AXL_:239, and Z_WT_ proteins were successfully labeled with DyLight-755 (Fig. S4A). In nude mice without tumor transplantation, DyLight-755-labeled Z_AXL_:239 affibody molecules were distributed throughout the body of nude mice within 30 min of injection and were almost completely excreted by the kidneys by 48 h after injection (Fig. S4B).

**Fig. 4:**
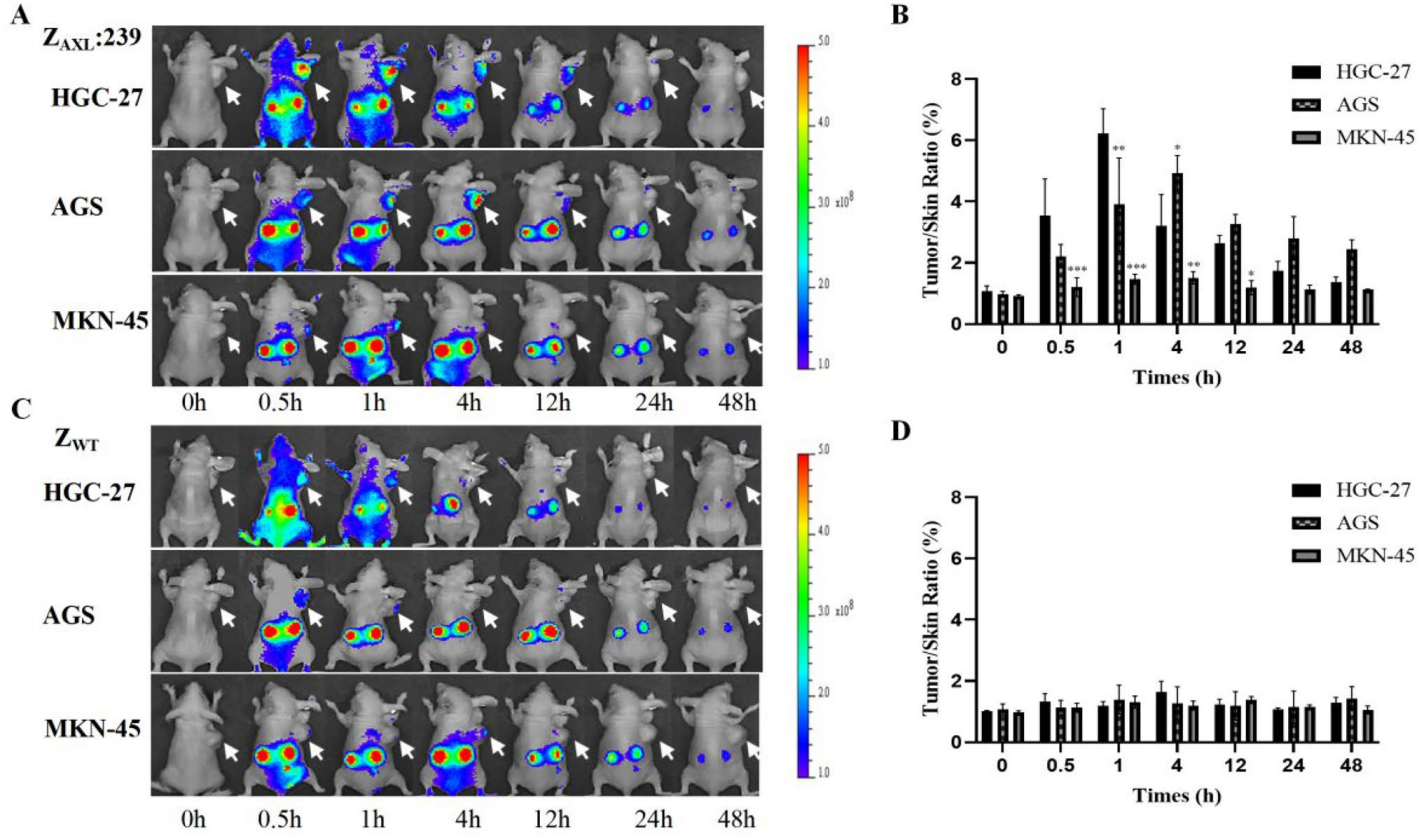
Tumor-targeted imaging in GC-bearing mice models of the Z_AXL_:239 affibody molecules. **A**, **C** Tumor-targeted imaging in model mice of the Dylight-755-labeled Z_AXL_:239 (**A**) or Dylight-755-labeled Z_WT_ (**C**). Tumor-bearing nude mice were generated with gastric cancer cell lines HGC-27, AGS, MKN-45. **B**, **D** Tumor/skin ratios were calculated at various time points post-injection of the indicated agents in HGC-27, AGS, and MKN-45 tumor-bearing mice. *p < 0.05; **p < 0.01; ***p < 0.001

The tumor-targeting ability of Z_AXL_:239 were then evaluated in nude mice bearing tumor xenografts by scanning with a NIR imaging device and quantifying fluorescence intensity. In the HGC-27 and AGS xenograft nude mouse model, we observed that high-contrast fluorescent signals appeared at the tumor site 30 min after injection, peaked at 4 h, and persisted for 24 h. As expected, no specific fluorescent features were observed in the MKN-45 xenograft tumor, and Dylight-755-labeled Z_WT_ protein showed no tumor-specific signal (Fig. 4A–D).

### Cell viability inhibition efficacy of Z_AXL_:239 affibody

**Fig. 5:**
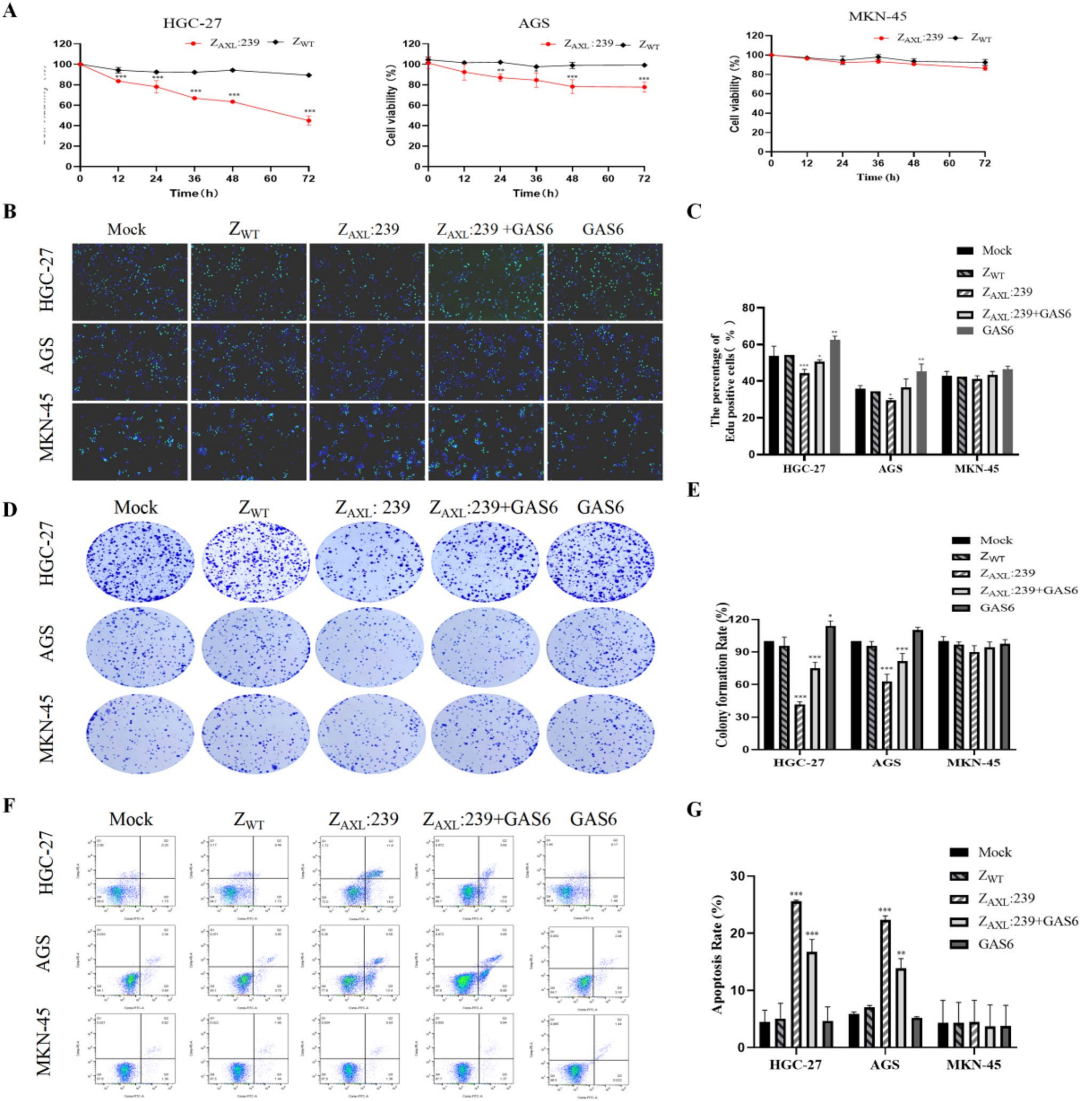
In vitro efficacy of Z_AXL_:239 affibody molecules. **A** CCK-8 assays were performed to determine the viability of gastric cancer cells treated with 10μM Z_AXL_:239 affibody molecules for the indicated times. The viability of AXL-positive cells (HGC-27, AGS) decreased with increasing incubation time with Z_AXL_:239 compared to the Z_WT_ control, whereas AXL-negative cells (MKN-45) treated with Z_AXL_:239 for the same times remained fully viable. **B**, **C** EdU staining (**B**) and relative EdU-positive cell ratios (**C**) for cells (HGC-27,AGS and MKN-45) treated with 10 μM Z_AXL_:239 affibody molecules or Z_WT_ control for 24 h. The values are shown as the mean ± SD in three wells. **D**, **E** colony formation (**D**) and relative colony formation ratios (**E**) for cells (HGC-27,AGS and MKN-45) treated with 5 μM Z_AXL_:239 affibody molecules or Z_WT_ control for 2 weeks. **F**, **G** apoptosis by flow cytometry (**F**) and relative percentage of early and late apoptosis (**G**) for cells (HGC-27,AGS and MKN-45) treated with 10 μM Z_AXL_:239 affibody molecules or Z_WT_ control for 24 h. *p < 0.05; **p < 0.01; ***p < 0.001

As shown in Fig. S4, we found that the viability of HGC-27 and AGS cells decreased with increasing concentrations of Z_AXL_:239. Data-processing results showed that the half-maximal inhibitory concentration (IC50) of cell viability with Z_AXL_:239 was 3.983 ± 0.754 μM for HGC-27 cells, 6.841 ± 0.763 μM for AGS cells, and greater than 20 μM for MKN-45 cells. The concentration of Z_AXL_:239 affibody molecules, 10µM, was selected for further investigation based on the IC50 values. Z_AXL_:239 significantly declined the viability of AXL-overexpression cells in a time-dependent manner, whereas GAS6 partially blocked the efficacy of Z_AXL_:239 (Fig. 5A). As expected, the Z_WT_ affibody did not affect the GC cells tested.

In addition, incubate of HGC-27 and AGS cells with Z_AXL_:239 affibody molecules reduced EdU staining and inhibited DNA replication (Fig. 5B,C) and significantly inhibited colony formation, whereas GAS6 significantly promoted colony formation in AXL-positive cell lines (Fig. 5D,E).

### Z_AXL_:239 affibody facilitates cell apoptosis

The apoptosis kit was then used to monitor the promoting effects of Z_AXL_:239 molecules on cell apoptosis by flow cytometry. The percentage of early and late apoptosis in HGC-27 and AGS cells increased after incubated with Z_AXL_:239 molecules compared with Z_WT_. At the same time, it has no significant effect on AXL-negative GC cell lines (Fig. 5F). The results of this flow-through study support that Z_AXL_:239 molecules can significantly promote apoptosis in AXL positive GC cells.

### Z_AXL_:239 downregulates signal transduction pathway in GC cells

**Fig. 6:**
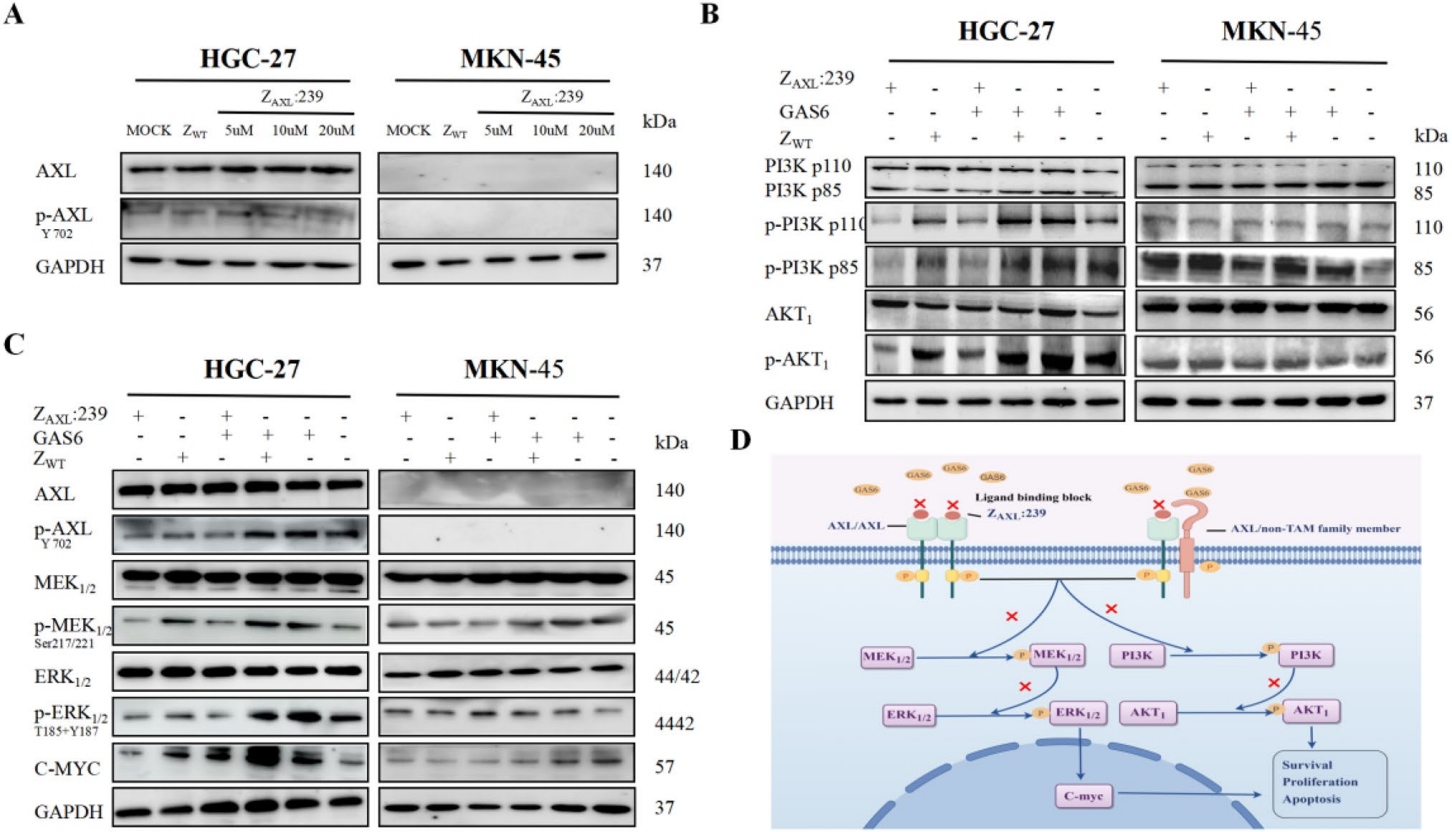
The potential mechanism of action of Z_AXL_:239 affibody molecules on gastric cancer cells. **A** The effect of different concentrations of Z_AXL_:239 affibody molecules on p-AXL^Y702^ by interacting with HGC-27 and MKN-45 cells for 36h. **B**, **C** p-AXL^Y702^, p-PI3K^p110/85^, p-AKT_1_, p-MEK_1/2_^Ser217/Ser221^, p-ERK_1/2_^Thr202/Thr204^ and C-myc were downregulated in a dose-dependent manner (**A**) by Z_AXL_:239 affibody treatment for 36h in HGC-27. Z_WT_ and medium groups were used as controls. GAPDH was used as internal reference standard. **D** Schematic of the potential model of Z_AXL_:239 blocking the AXL-mediated signaling pathways involving MAPK and PI3K/AKT_1_ pathway

To investigate whether Z_AXL_:239 by blocking AXL affects the PI3K/AKT1 and MEK/ERK/C-myc pathway, we used Western blot analysis, which showed that p-AXL^Y702^ levels decreased in Z_AXL_:239-treated HGC-27 cells (Fig. 6A, S5A,B). According to the above results, treated with 10-µM Z_AXL_:239 for 36 h were selected to further experiments. The results showed that HGC-27 cells incubated with Z_AXL_:239 downregulated the levels of PI3K and AKT1, as well as phospho-MEK_1/2_^Ser217/Ser221^, phospho-ERK_1/2_^Thr202/Thr204^ in the MEK/ERK pathway and the transcriptional regulator c-myc, while blocking the promoting effect of the ligand gas6 (Fig. 6B, S5C). Similar results were observed after silencing AXL expression (Fig. S5D). At the same time, MKN-45 cells incubated with Z_AXL_:239 did not produce similar results. The Schematic representation of how Z_AXL_:239 blocks AXL-mediated signaling through the PI3K/AKT1 and MEK/ERK/C-myc pathway pathway (Fig. 6C). In conclusion, Z_AXL_:239 inhibited the proliferation and promoted the apoptosis of HGC-27 and AGS cells by down-regulating the PI3K/AKT1 and MEK/ERK/c-myc signaling pathway.

### In vivo therapeutic efficacy of Z_AXL_:239

**Fig. 7:**
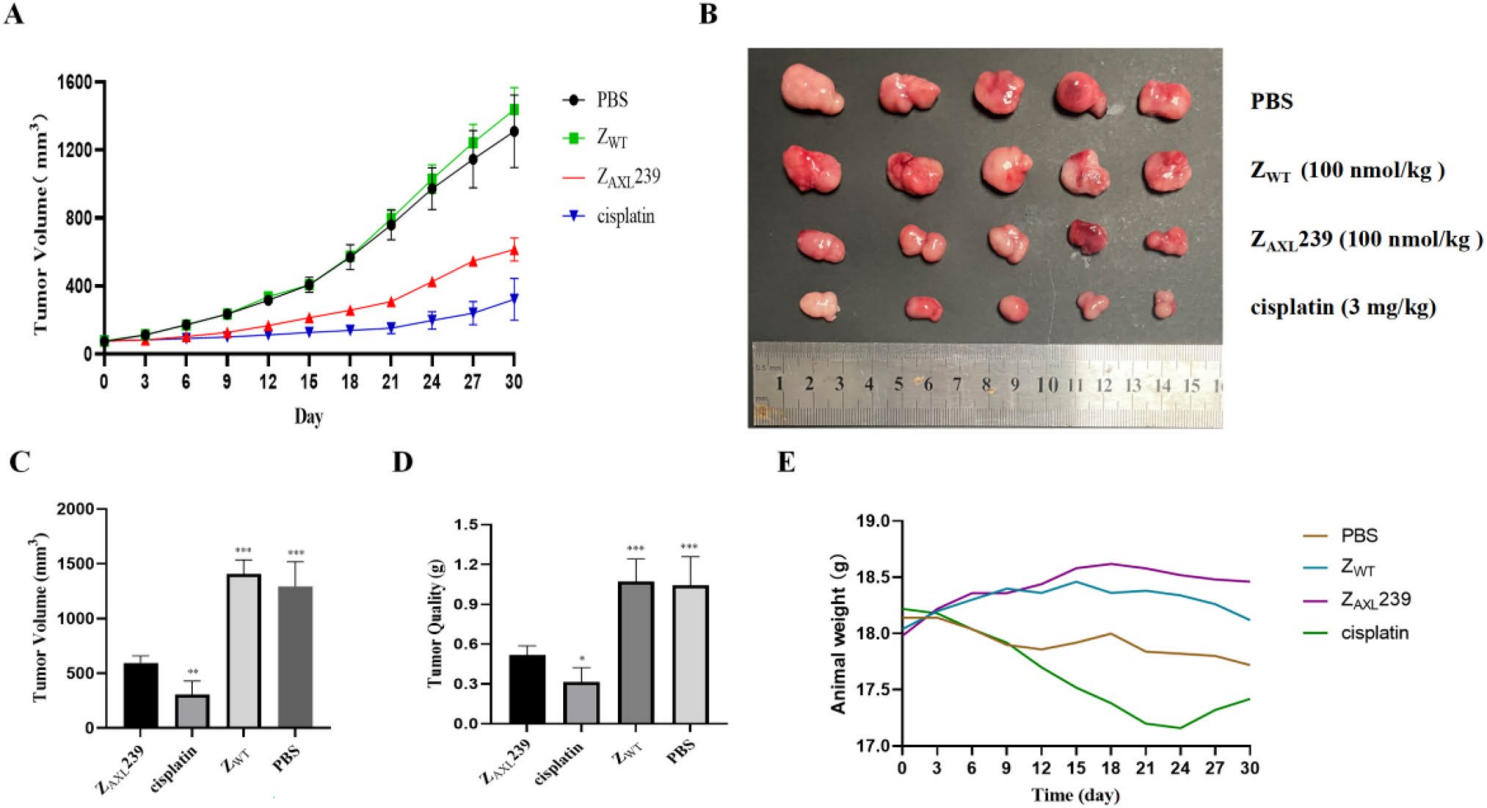
Therapeutic efficacy of Z_AXL_:239 in GC-bearing nude mice. **A**, **B** Tumor-bearing mice were randomly divided into four groups, then different treatments (PBS, Z_WT_, Z_AXL_:239, and cisplatin) were administered every 3 days, and tumor volume was measured (*n* = 5). Mice model of subcutaneous grafted tumor constructed from GC cells HGC-27. **C** Tumor volume and **D** tumor weight. **E** Body weight of HGC-27 tumor-bearing mice. Data are expressed as mean ± SD. *p < 0.05; **p < 0.01; ***p < 0.001

The in vivo antitumor effect of the Z_AXL_:239 affibody was evaluated by measuring tumor growth in mice bearing HGC-27 xenografts. As shown in Fig. 7A, tumor growth in HGC-27 bearing mice in the treated groups (Z_AXL_:239 and cisplatin) was slow during the treatment period. More critically, treatment with Z_AXL_:239 signficantly reduced tumor growth much more inhibitory than the control Z_WT_ (Fig. 7B,D). As expected, the Z_WT_ and PBS did not show any anti-tumor effect. No significant side effects of Z_AXL_:239 drug treatment were observed in mice based on dietary effects and body weight (Fig. 7E). Until the end of this experiment, we did not see any mice die from the treatment agents during the observation period. In conclusion, our results provide the first evidence that Z_AXL_:239 may be a useful targeted therapeutic agent specific for AXL positive GC tumors.

## Discussion

Although targeted therapies in GC has developed greatly, the prognosis of patiens is not satisfactory. Affibody molecules are new class of affinity proteins [9–11], attempts have been made to develop them for various clinical needs, including molecular imaging of tumors, blockade of receptor signaling, or delivery of toxic payloads in tumor therapy. Due to their small size, less than 1/20 the size of antibodies, affibody molecules enable efficient tumor penetration and rapid tumor localization. In this study, three novel affibody molecules (Z_AXL_:4, Z_AXL_:239, and Z_AXL_:361) were eluted from the constructed library using phage display library technology and underwent prokaryotic expression and purification. Then, we found that Z_AXL_:239 affibody molecules had the highest affinity for recombinant AXL protein by ELISA assay with high protein expression efficiency and were selected for further experiments. In the real-time biosensing signal assay, the Z_AXL_:239 affibody molecules showed a 10^5^-fold higher affinity for AXL. Meanwhile, immunofluorescence co-localization experiments and immunoprecipitation experiments confirmed the the existence of interactions between the Z_AXL_:239 molecules and native AXL proteins, further demonstrating the binding specificity of Z_AXL_:239 with targeted AXL proteins. The above results demonstrate that Z_AXL_:239 has good binding affinity and specificity for AXL target proteins, having potential use as a powerful imaging molecular probe.

In vivo molecular imaging can provide a global view, including potential metastatic lesions, great importance for the diagnosis of many diseases, guiding treatment and assessing prognosis [34]. However, the technique is difficult to use for the specific detection of gastrointestinal tumors due to the lack of specific targets or corresponding imaging agents with high specificity and affinity [35]. Currently, molecular imaging of gastrointestinal tumors is mainly through metabolic markers such as 18F-fluorodeoxyglucose PET, which takes advantage of increased glucose metabolism in tumors, does not identify cancer-specific targets. Specific molecular imaging and targeted tumor therapies depend primarily on their affinity tools, such as monoclonal antibodies and fragments. Compared to mAb, affibody molecules have low molecular weight, high permeability, no immunogenicity issues and easy clearance, which are favorable properties for molecular diagnostic imaging [36]. Until now, the PET affibody tracer (ABY-025) has been useful in the diagnosis of HER2-overexpressing tumors [37–39]. Using TCGA public datasets and immuno-histochemistry of clinical tissues, we demonstrated that AXL is upregulated in GC patients, making it a suitable target for molecular diagnostic imaging. In our animal NIR imaging experiments, following injection of DyLight 755-labeled Z_AXL_:239 protein into HGC-27 and AGS nude mice bearing tumor xenografts, Z_AXL_:239 rapidly localized to the tumor at 1 h, accumulated in large amounts at 12 h, and was then predominantly cleared by the kidney and liver within 48 h. Taken together, the Z_AXL_:239 affibody molecules as imaging image probes can rapidly penetrate tissues and specifically bind to corresponding tumors, thereby improving the contrast and specificity of molecular imaging images.

Molecular targeted therapy is an essential class for GC treatment, especially for metastatic and unresectable gastric cancer patients. Currently, mAbs such as trastuzumab have made some cancer progress in clinical practice for GC [40]; the stability, immunogenicity, and size limitations of mAbs have prompted researchers to seek solution strategies. Affibody molecules are expected to become agents with remarkable affinity and specificity for clinical molecular targeted therapy, such as HPV16E7 and HER-2 affibody molecules have potent biological activity used in targeted therapies [41–43]. In this study, we found that AXL was upregulated in GC patients clinical tissues, and its high expression was a prognostic factor for poor overall survival (OS). GAS6/AXL signaling plays an essential role in proliferation, drug resistance, and inhibition of apoptosis [25, 27–29]. We treatment with Z_AXL_:239 affibody molecules in a time- and concentration-dependent manner significantly reduced cell viability and promoted induced cell apoptosis in AXL-positive GC cell. More critically, treatment with Z_AXL_:239 significantly reduced tumour growth in the mice bearing AXL-positive GC cell xenografts model and no significant side effects of Z_AXL_:239 drug treatment were observed.

A study of an AXL homodimerization and heterodimerization chimeric receptor revealed that Tyr821 and Tyr866 of phosphorylated AXL activate the PI3K/AKT1 and MEK/ERK cascade [44]. In our research, the expression of p-AXL^Y702^, p-PI3K, p-AKT1, p-MEK_1/2_^Ser217/221^, p-ERK_1/2_^T185+Y187^, and the downstream factor c-Myc were significantly reduced after Z_AXL_:239 treatment as shown. In short, the above results show that Z_AXL_:239 affibody molecules may inhibit GC tumor cell proliferation by blocking GAS6 binding to the extracellular region of AXL, inhibiting activation of downstream signaling factors, inhibiting cell viability, and promoting apoptosis [45].

In brief, we constructed an AXL binding affibody protein (Z_AXL_:239) with high affinity and specificity for AXL’s extracellular membrane structural domains. In vivo, Z_AXL_:239 protein rapidly localized and accumulated in tumors of a tumor xenograft nude mouse model. The Z_AXL_:239 affibody significantly reduced cell viability and inhibited tumor proliferation by inhibiting the phosphorylation levels of the PI3K/AKT1 and MEK/ERK pathway in mice model. Still, further studies are necessary to determine the in vivo antitumor effects and clinical applicability of Z_AXL_:239 affibody molecules. The AXL-binding affibody molecules (Z_AXL_:239) is a potent molecular imaging and targeted therapeutic agent that may be useful in the diagnosis and targeted therapy of GC.

## Materials and methods

### Bioinformatics analysis

Bioinformatics data, including transcriptomic data, patient clinical characteristics and prognostic data, were collected from the Cancer Genome Atlas database (https://portal.gdc.cancer.gov/) and the Kaplan–Meier Plotter website database (http://kmplot.com/).

### Immunohistochemical analysis of microarrays

GC and para-cancer tissue microarrays were purchased from Shanghai Zhuo Li Biotechnology Co. Tissue microarrays were heated in 10 mmol/L and pH 6.0 of sodium citrate buffer for antigen retrieval and then immunostained with rabbit anti-AXL monoclonal primary antibody (1:100) and sheep anti-rabbit HRP secondary antibody (1:500), followed by microscopic reading of sections and photographic documentation. AXL expression in tissues was independently reviewed and scored by two pathologists based on the immunohistochemical results.

### Construction of phage display library

Affibody phage libraries were prepared as described in previous articles [17]. A recombinant vector library with a library size of 1 × 10^9^ and 100% diversity was obtained, and potential affibody molecules that could specifically bind the AXL recombinant protein were screened using phage display technology.

### Molecular docking simulation

The full-length amino acid sequence of AXL (GenBank: AAH32229.1) was searched in the NCBI database. The crystal structure of the AXL-Gas6 complex (PBD:2C5D) was found from the PBD database (https://www.rcsb.org/), and the interaction region between AXL and Gas6 was analysed by pymol software [46].

### Design the combined AXL for affibody screening

In this report, 22–96 amino acid positions of AXL were selected according the previous results. Codon-optimized nucleotide sequences were synthesized by Shanghai Sangon Biotech Co, which were then cloned into the pET-21a( +) vector and transformed into E. coli BL21 for expression.

### Screening of AXL-binding affibody molecules

Briefly, we constructed and prokaryotically expressed the recombinant protein AXL for screening. Details of the screening and washing methods can be found in a previously published article [43].

### Expression and purification of ZAXL affibody molecules

Based on the screening results, the Z_AXL_:4, Z_AXL_:239 and Z_AXL_:361 affibody were identified for expression. The AXL-binding and Z_WT_ affibody were cloned into the pET-21a( +) expression vector with a C-terminal His-tag, then transformed into E. coli BL21 (DE 3) cells and the expression and purification details were as described in previous articles [17].

### Surface plasmon resonance analysis (SPR)

The AXL-recommended protein was diluted as a ligand (pH 4.5) and immobilised on the carboxylated glucan surface of the CM-5 sensor chip. Different concentrations of Z_AXL_:239 and Z_WT_ were sequentially injected onto the surface of the chip. The results of binding dissociation data were fitted globally using a 1:1 Langmuir binding model and analysed using Biacore software (BIA evaluation 4.0.1 DC, USA).

### Cell culture

All cell lines were obtained from the National Identification Cell Bank Shanghai Cell Bank. HGC-27 cells were grown in DMEM medium, AGS and MKN-45 cells were grown in RPMI-1640 medium supplemented with 10% fetal bovine serum, and cultured in an incubator at 37 °C and 5% CO_2_.

### Immunofluorescence detection

GC cells were inoculated into 12-well plates at a density of 1 × 10^5^ cells/well, and then incubated with fresh medium containing 5 μmol/L Z_AXL_:239 or Z_WT_ for 4 h at 37 °C. For specific experimental methods, please refer to the published literature [47]. Finally, an Upright Microscopes fluorescence microscope (CI-L, Japan) was used for imaging and photographic documentation.

### Co-immunoprecipitation assay

Cells were harvested, lysed, and incubated for 6 h with the addition of Z_AXL_:239 or Z_WT_ affibody. Anti-His labeled rabbit antibody was then added and incubated for overnight. Protein A/G plus agarose was subsequently added to form protein complexes for 3 h. After several washes with PBS, the protein–agar complexes were analysed by Western blotting.

### Nude mouse animal models

The subcutaneous transplantation tumour model was established by subcutaneous injection of 5 × 10^6^ cells into the right axilla of female nude mice (Shanghai Slaughter Laboratory Animal Co, BALB/c, 4–5 weeks). All institutional and national guidelines for the care and use of laboratory animals were followed, and approved by the Ethics Committee of Wenzhou Medical University (Ethics No. wydw2023-0181).

### Cell viability inhibition assay

All cells were inoculated into 96-well plates at 5 × 10^3^ cells/well and then incubated with increasing concentrations of Z_AXL_:239 or Z_WT_ for 72 h and then scored using the Cell Counting Kit-8. Absorbance was measured at 450 nm using an enzyme marker (TECAN, Infinite M200 pro, Switzerland). Half-maximal inhibitory concentrations were calculated from the absorbance data. Concentration of 10 μm was then selected to assess the efficacy incubated with Z_AXL_:239 affibody for different time periods.

### EdU cell proliferation assay

The effect of the Z_AXL_:239 on cell DNA replication was evaluated using an EdU labeling/detection kit. The procedure is similar to that described above for immunofluorescence detection. Cellular DNA was stained by incubation with anti-EdU working solution for 30 min, followed by incubation with Hoechst 33,342 to stain nuclei. Five randomly selected fields of view were used to calculate the percentage of EdU-positive cells.

### Colony formation assay

Cells were digested, centrifuged, and inoculated into 6-well plates at 1 × 10^3^ cells/well density and then treated with 5μM Z_AXL_:239. After two weeks, cells were rinsed with PBS, fixed in methanol at  – 20 degrees Celsius, stained with 0.1% crystalline violet staining solution (Beyotime, C0121, Chian) and colonies were counted.

### Cell apoptosis analysis

Cells were harvested by incubation with Z_AXL_:239 affibody for 24 h. For further procedures, please refer to the manufacturer’s instructions. Apoptotic cells were detected by incubation with AnnexinV/propidium iodide (PI) and Fluorescein 5-isothiocyanate (FITC). Samples were collected using a FACS Calibur flow cytometer (BD, FACS CantoII, USA), and data were analysed using FlowJo v10.8 software.

### Western blot analysis

After centrifugation to collect the cells, they were completely lysed with pre-cooled lysate to extract total cell proteins. Protein samples were separated by SDS-PAGE electrophoresis, and after blotting the proteins onto PVDF membranes, immersed in blocking buffer (5% BSA) for 1 h and shaken overnight at 4 °C with primary antibodies. The samples were then incubated with goat anti-rabbit/mouse secondary antibodies for 2 h at 25 °C. After the addition of ECL chemiluminescent solution, exposure and data storage were performed in a BIO-rad imaging device.

### Vivo antitumor efficacy of Z_AXL_:239 in mice models

The establishment of HGC-27 xenograft mouse tumor models was described above. The tumor sizes and body weights of the nude mice were recorded every 3 days until the end of the experiments. When the tumor volume reached 50 ~ 100 mm^3^, the mice were injected with 100 μL of Z_AXL_:239 (100 nmol/kg), Z_WT_ (100 nmol/kg), cisplatin (4 mg/kg) or PBS via the tail vein 7 times every 3 days. The therapeutic efficacy and systematic toxicity of the affibody proteins were evaluated by measurements of tumor volume and body weight. On day 30, all mice were sacrificed, and tumors from mice in the above four groups were carefully removed, weighed, and stored at  – 80 °C.

### Statistic analysis

Values are expressed as mean + standard deviation (SD). Statistic differences between groups were analysed by unpaired *t* test and considered statistically significant with a p value of less than 0.05. All data analyses were performed with SPSS 25.0.

## Supplementary Information

Below is the link to the electronic supplementary material.

Supplementary file1 (DOCX 39140 KB)
