# Supplementary material for: Novel affibody molecules targeting the AXL extracellular structural domain for molecular imaging and targeted therapy of gastric cancer
Source: Gastric Cancer. 2024 Dec 7;28(2):174–86. doi: 10.1007/s10120-024-01568-5 (PMC11842530; doi:10.1007/s10120-024-01568-5)
Supplement: Supplementary file 1 — Supplementary file1 (DOCX 39140 KB) [file 10120_2024_1568_MOESM1_ESM.docx]

Table S1:Screening for subgroups of AXL expression in gastric cancer populations.


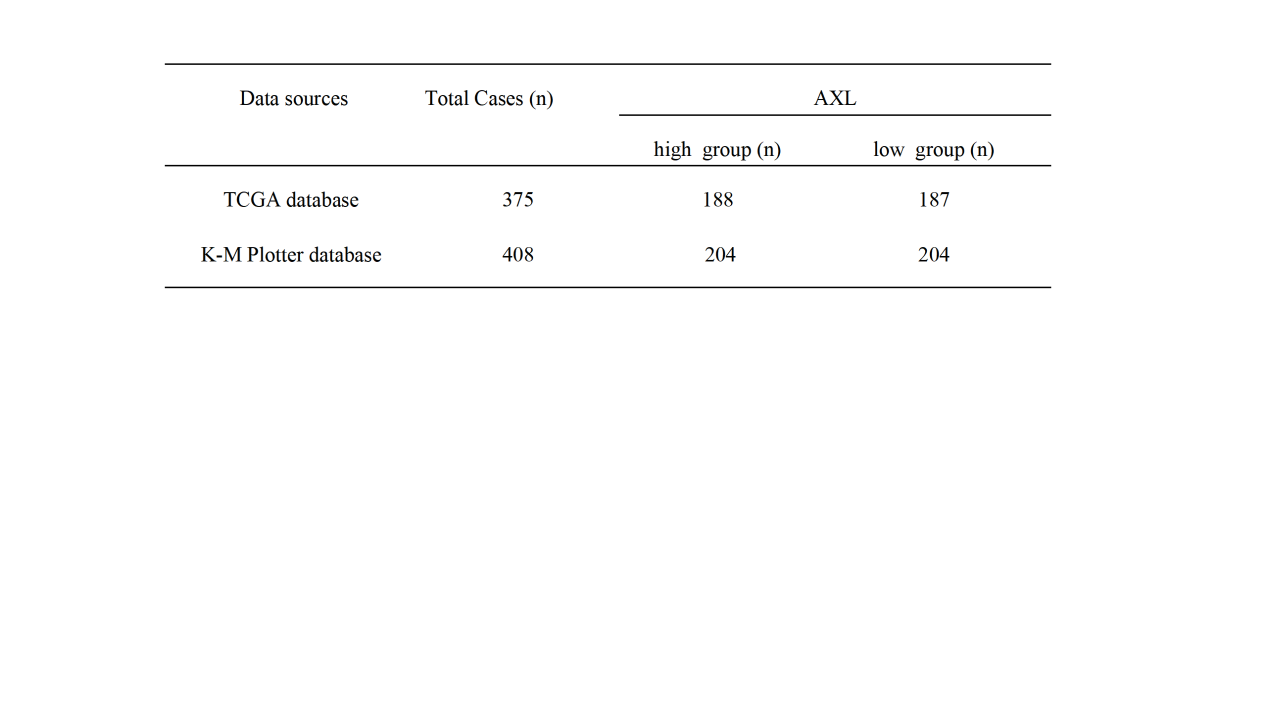


Abbreviations: TCGA, the Cancer Genome Atlas database; K-M Plotter database, Kaplan-Meier Plotter website database.

Table S2: The correlation of AXL expression with clinicopathologic characteristics of GC patients.


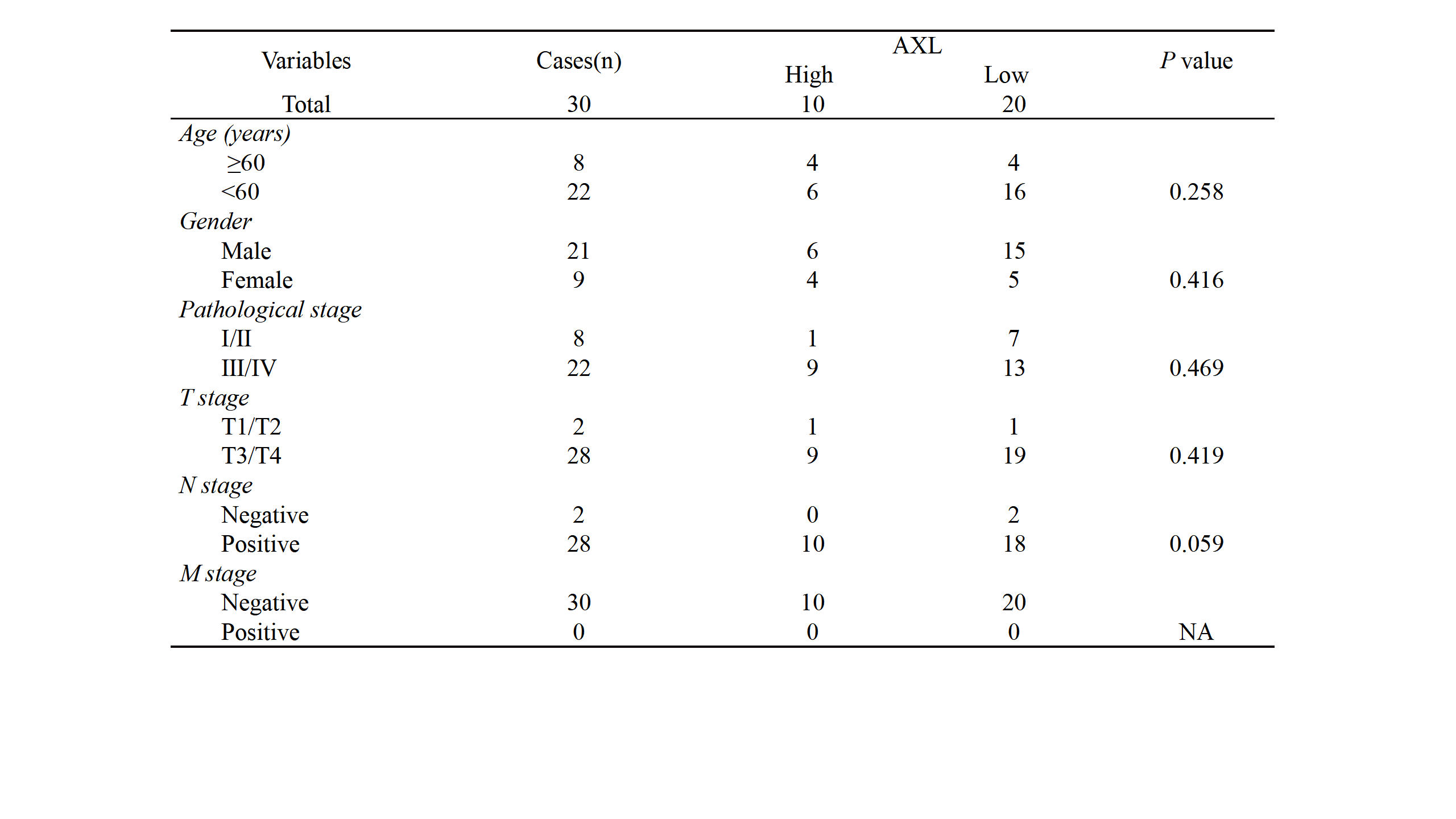


Table S3: Cox regression analysis of AXL expression as survival predictor.


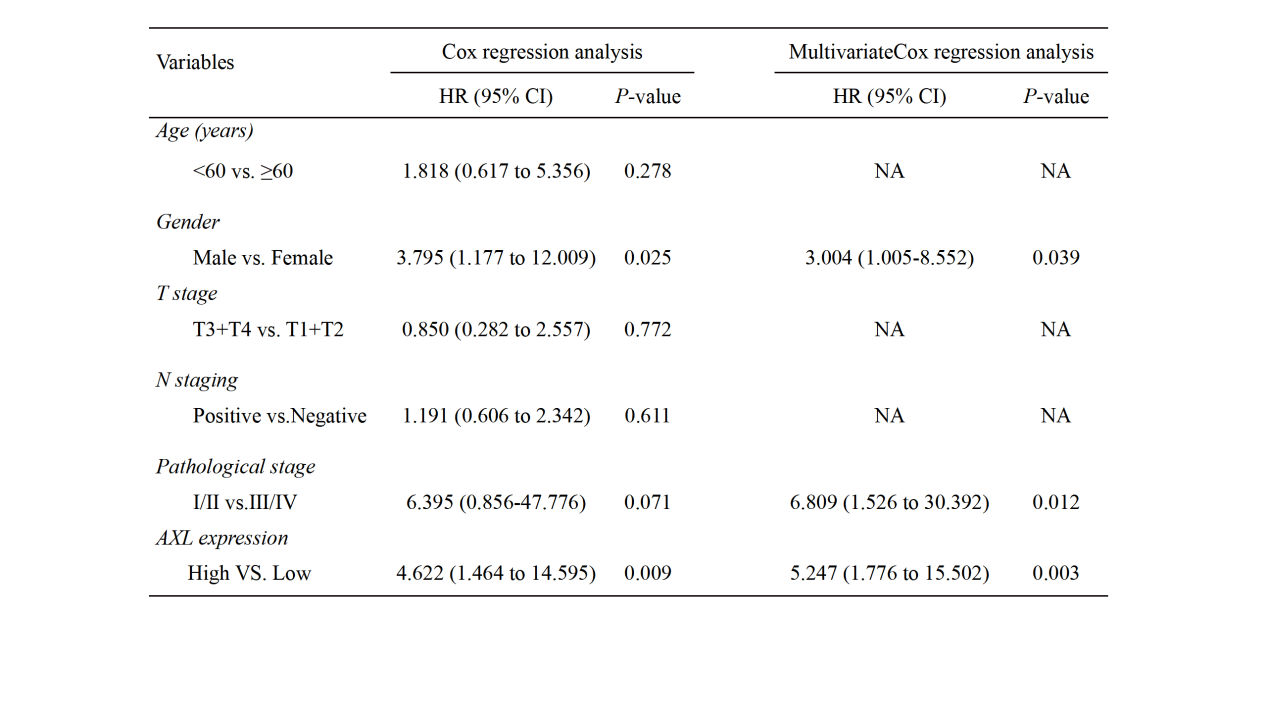


Figure S1: Schematic diagram of the screening elution procedure for specific affibody molecules.


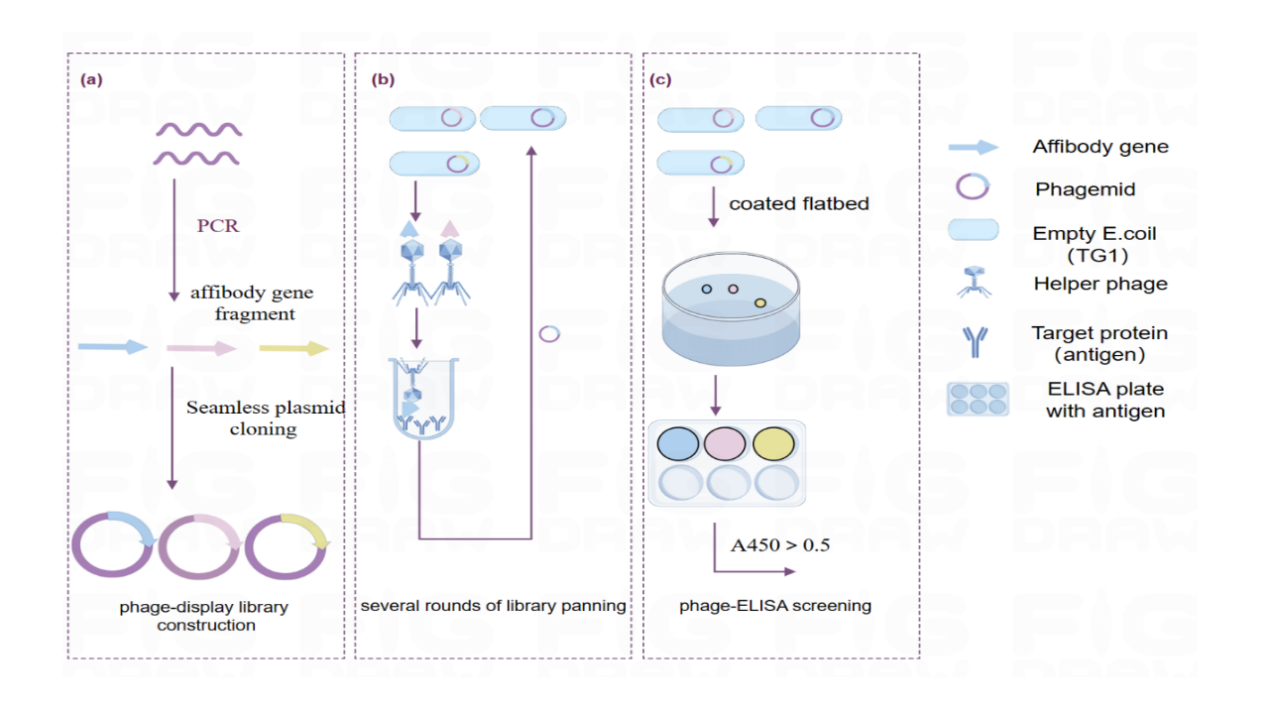


Figure S2: Preparation of recombinant proteins of AXL and ligand Gas6.


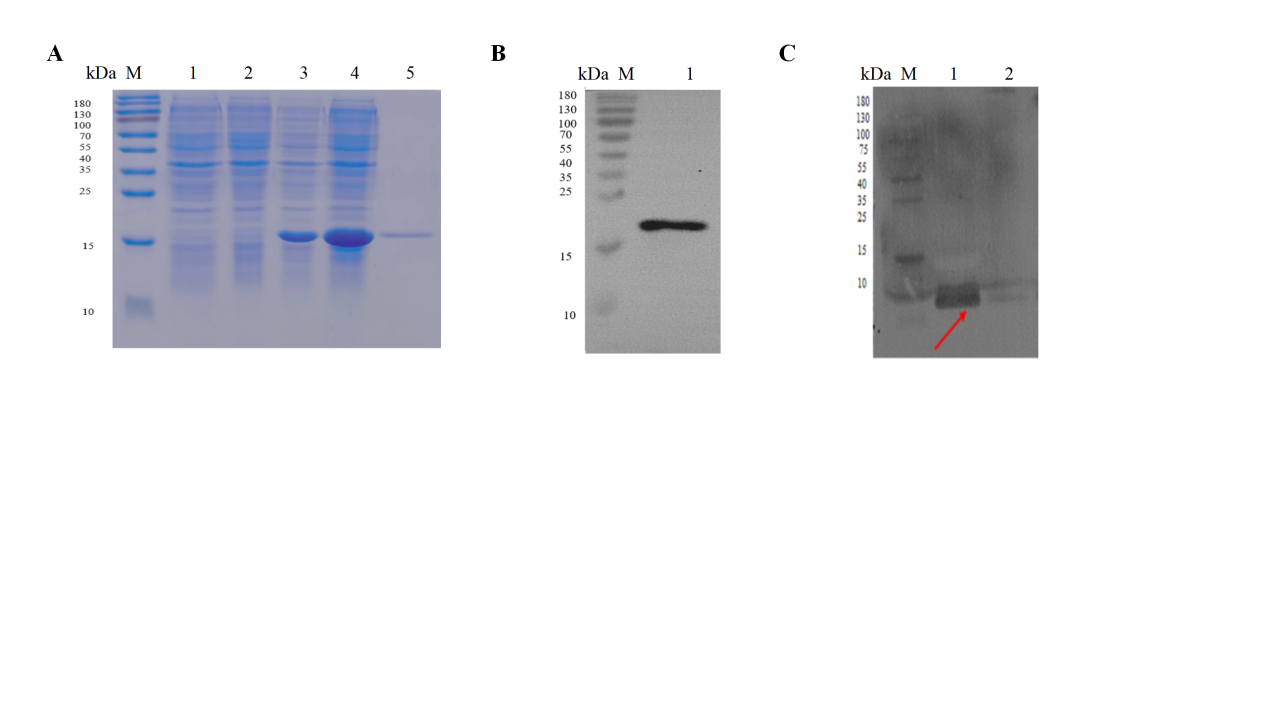


(A, B) Gas6 recombinant protein Tris-Tricine -SDS-PAGE gel electrophoresis results: M: Marker ;1: E.coli.BL21 (DE3); 2:pET21a(+)/E.coli.BL21 (DE3); 3: pET21a(+)/Gas6 E.coli.BL21 ( DE3) before induction; (4): pET21a(+)/Gas6 E. coli.BL21 (DE3) induced by 1 mM IPTG for 6 h; (5): purified protein GAS6 were analyzed by SDS-PAGE and confirmed by Western blotting. (C)The recombinant protein AXL binds specifically to the recombinant protein Gas6. Western Blot results: M: Marker ;1:AXL recombinant protein; 2:IL-4 recombinant protein.

Figure S3: Biodistribution imaging of Dylight-755-labelled Z_AXL_:239 and Dylight-755-labelled Z_WT_ in nude mice model.


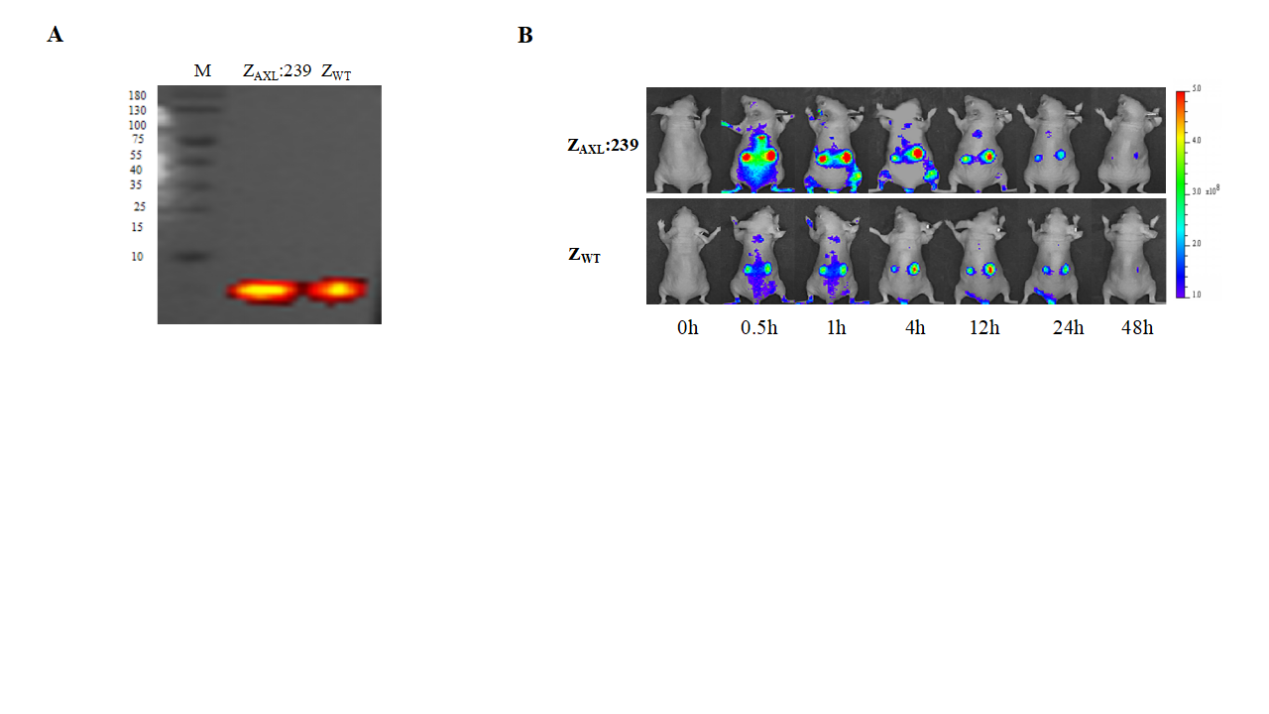


(A) Z_AXL_:239 and Z_WT_ proteins were labeled with dylight-755 dye and visualized by western blot followed using NIR spectroscopy. (B) Biodistribution imaging of Dylight-755-labelled Z_AXL_:239 and Dylight-755-labelled Z_WT_ in nude mice.

Figure S4: Half effective inhibitory concentration of Z_AXL_:239 affibody molecules on the viability of gastric cancer cell lines.


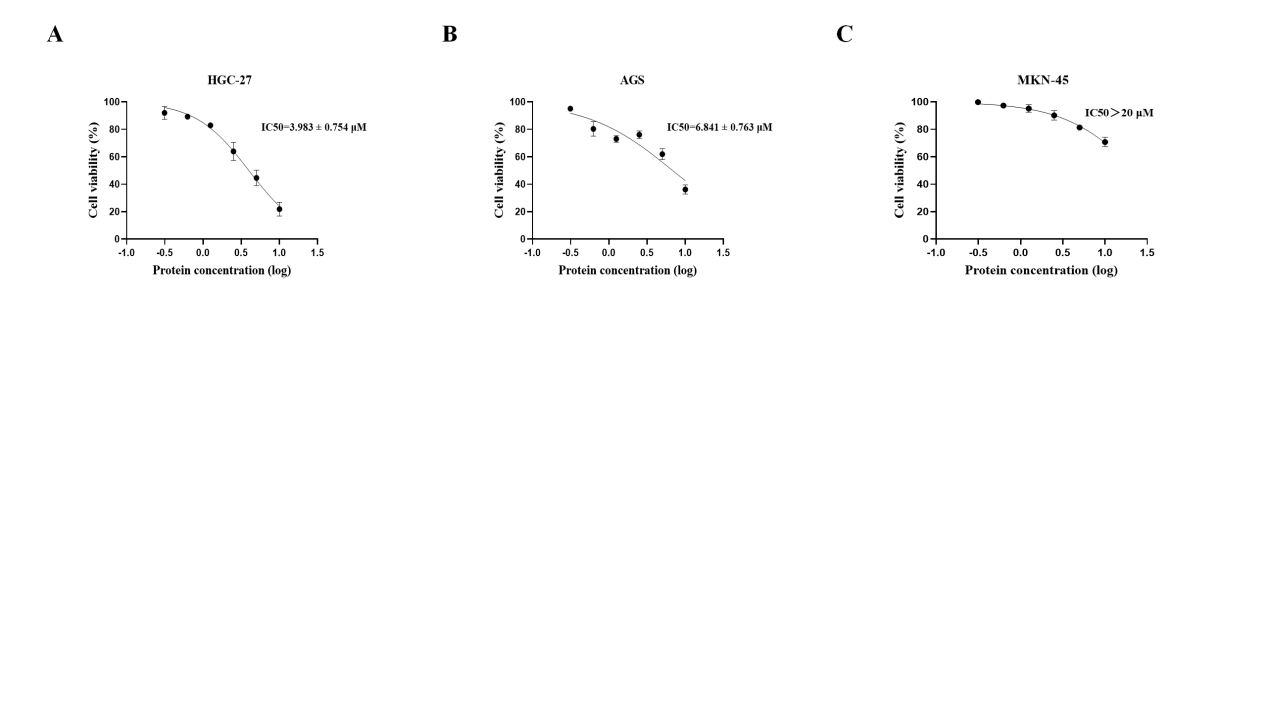


(A,C) the half-maximal inhibitory concentration of cell viability was 3.983 ± 0.754μM for HGC-27 cells (A), 6.841 ± 0.763μM for AGS cells (B), and greater than 20μM for MKN-45 cells (C).

Figure S5: Effect of Z_AXL_:239 affibody molecules and GAS6 recombinant protein with different action times on AXL phosphorylation in GC cells.


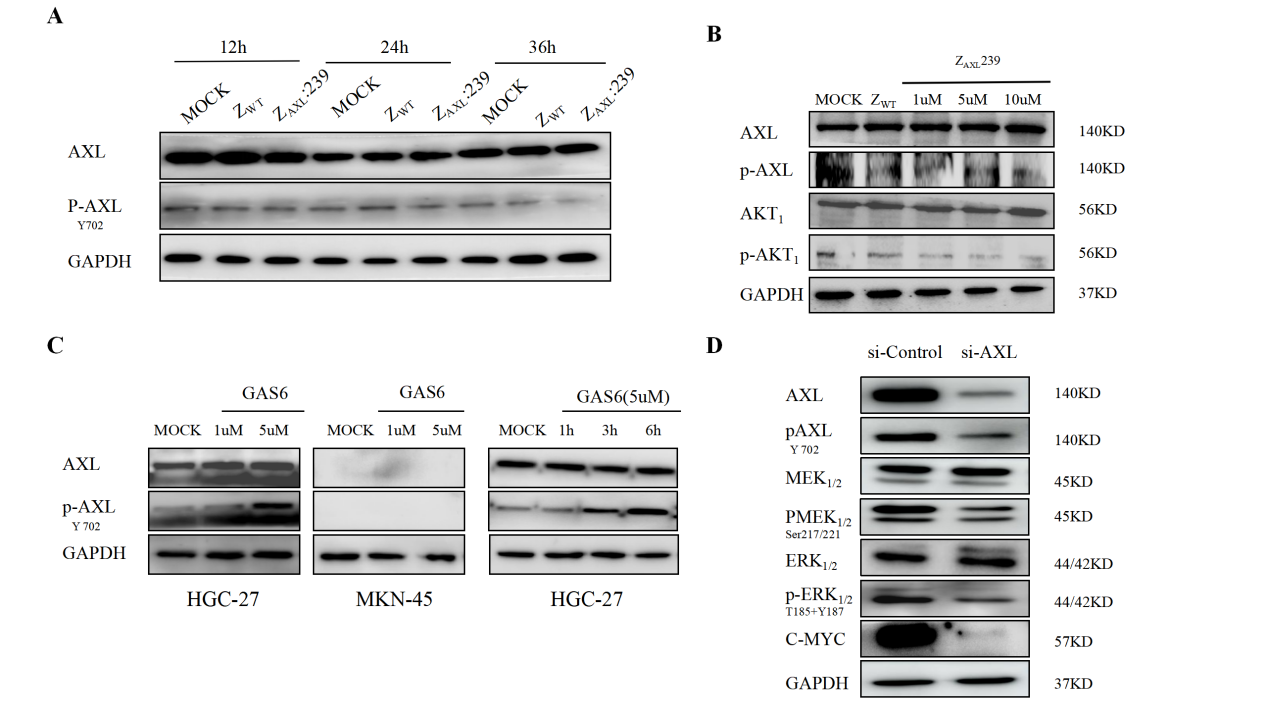


(A)Effects of different periods of Z_AXL_:239 affibody molecules on the p-AXL^Y702^ in HGC-27. (B) Effects of different concentrations of Z_AXL_:239 affibody molecules on the p-AXL^Y702^ and pAKT_1_ in HGC-27. (C) Effects of different concentrations of activator GAS6 recombinant protein acting for 6h on p-AXL^Y702^ in HGC-27 and MKN-45. (D) Effects of si-AXL interference with AXL proteins on MAPK downstream signaling factors.
